# Environmental DNA (eDNA) Sampling Improves Occurrence and Detection Estimates of Invasive Burmese Pythons

**DOI:** 10.1371/journal.pone.0121655

**Published:** 2015-04-15

**Authors:** Margaret E. Hunter, Sara J. Oyler-McCance, Robert M. Dorazio, Jennifer A. Fike, Brian J. Smith, Charles T. Hunter, Robert N. Reed, Kristen M. Hart

**Affiliations:** 1 US Geological Survey, Southeast Ecological Science Center, Gainesville, Florida, United States of America; 2 US Geological Survey, Fort Collins Science Center, Fort Collins, Colorado, United States of America; 3 University of Florida, Department of Wildlife Ecology and Conservation, Gainesville, Florida, United States of America; 4 University of Florida, Institute of Food and Agricultural Sciences, Horticultural Sciences, Gainesville, Florida, United States of America; 5 US Geological Survey, Southeast Ecological Science Center, Davie, Florida, United States of America; Central Michigan University, UNITED STATES

## Abstract

Environmental DNA (eDNA) methods are used to detect DNA that is shed into the aquatic environment by cryptic or low density species. Applied in eDNA studies, occupancy models can be used to estimate occurrence and detection probabilities and thereby account for imperfect detection. However, occupancy terminology has been applied inconsistently in eDNA studies, and many have calculated occurrence probabilities while not considering the effects of imperfect detection. Low detection of invasive giant constrictors using visual surveys and traps has hampered the estimation of occupancy and detection estimates needed for population management in southern Florida, USA. Giant constrictor snakes pose a threat to native species and the ecological restoration of the Florida Everglades. To assist with detection, we developed species-specific eDNA assays using quantitative PCR (qPCR) for the Burmese python (*Python molurus bivittatus*), Northern African python (*P*. *sebae*), boa constrictor (*Boa constrictor*), and the green (*Eunectes murinus*) and yellow anaconda (*E*. *notaeus*). Burmese pythons, Northern African pythons, and boa constrictors are established and reproducing, while the green and yellow anaconda have the potential to become established. We validated the python and boa constrictor assays using laboratory trials and tested all species in 21 field locations distributed in eight southern Florida regions. Burmese python eDNA was detected in 37 of 63 field sampling events; however, the other species were not detected. Although eDNA was heterogeneously distributed in the environment, occupancy models were able to provide the first estimates of detection probabilities, which were greater than 91%. Burmese python eDNA was detected along the leading northern edge of the known population boundary. The development of informative detection tools and eDNA occupancy models can improve conservation efforts in southern Florida and support more extensive studies of invasive constrictors. Generic sampling design and terminology are proposed to standardize and clarify interpretations of eDNA-based occupancy models.

## Introduction

Environmental DNA (eDNA) is increasingly being used for detection of rare and non-native species to inform management actions, especially when traditional field methods are inadequate [1–5]. Environmental DNA originates from cellular material shed by organisms (via skin, excrement, gametes etc.) into aquatic or terrestrial environments [4,6]. Improved methods of species detection and quantification of imperfect detection are vital to management of invasive species, since the effects of imperfect detection can result in an underestimation of the distribution of the species [2,3,7–10]. Further, the ability to detect non-native species at low densities or prior to establishment is critical for successful control and eradication efforts [1,11,12].

Schmidt et al. [10] used a three-level occupancy model to re-analyze eDNA detections of an invasive fungal pathogen (*Batrachochytrium dendrobatidis*), accounting for imperfect detection. Using this model, estimates of occurrence probability were 5–10% higher than estimates that did not account for the effects of field or laboratory eDNA detection errors. Despite improved methods, other eDNA studies have largely ignored the effects of imperfect detection when estimating occurrence [6,13,14]. Furthermore, these studies appear to have confounded variation in eDNA occurrence among samples (i.e., environmental heterogeneity) with variation in eDNA detection ˗ that is, a failure to observe eDNA in a sample was interpreted as a nondetection of eDNA, as opposed to an absence of eDNA in the sample. It is entirely possible for the eDNA of a species to be absent or present in samples collected from a location where the target species is present [14]. Standardized sampling design and occupancy model terminology are needed for consistent interpretation of occurrence and detection probabilities reported in eDNA surveys.

Environmental DNA detection and the implementation of occupancy models could improve management of invasive giant constrictor snakes in the Greater Everglades ecosystem (GEE) in southern Florida. Native to Southeast Asia, Burmese pythons (*Python molurus bivittatus or Python bivittatus) are* established and reproducing in Florida. They pose a significant threat to native prey species, including mammals, birds, and reptiles listed under the U.S. Endangered Species Act [15–18]. A number of mammal species appear to have suffered precipitous declines in the GEE due to predation by the Burmese python [18]. In addition to the Burmese python, the Northern African python (*P*. *sebae*; also known as the African rock python) and boa constrictor (*Boa constrictor*) are locally established along the western and southern fringes of Miami, respectively [19,20]. Reticulated pythons (*Malayopython reticulatus*) and both green (*Eunectes murinus*) and yellow (*E*. *notaeus*) anacondas are also found in the pet trade and are species of concern as potential invaders. Burmese pythons are considered semi-aquatic, spending much of their time near water, while anacondas are almost entirely aquatic [21]. Typically, pythons and boa constrictors use aquatic habitats facultatively. Free-ranging individuals of all three species have been found in Florida, but established populations have not yet been identified.

Burmese pythons are extremely cryptic and are believed to occupy thousands of square kilometers of mostly inaccessible habitats, making the development of detection methods difficult. Several tools for physical detection and control (including detector dogs, remote sensing, attractant traps, “Judas snakes,” etc.) have been tested for Burmese pythons in Florida, with varying degrees of success (K. Hart, personal communication). Due to the difficulty in visual detection in the Everglades ecosystem, only a single study has attempted to estimate detection probabilities using traditional visual searching or trapping tools. Using two types of traps, the capture rate was 0.05% per trap night (3 pythons captured in 5,935 trap nights) [22]. Standardized visual surveys resulted in no sightings, although two pythons were observed opportunistically.

Non-invasive monitoring of invasive snake species using eDNA could provide a decision-support tool for long-term management strategies through the estimation of occurrence and detectability using occupancy models. This approach also can assist in identifying newly-colonized areas, movement corridors, and potential pathways of dispersal. More precise information on the presence of invasive constrictors in imperiled ecosystems can inform spatiotemporal assessment of risk to imperiled native species (e.g., ground-dwelling birds, Key Largo woodrat (*Neotoma floridana smalli*), Florida panther (*Puma concolor*)), and potentially allow for targeted removal efforts prior to realization of major ecological and economic impacts [15–17]. Environmental DNA tools also could assist with short- or long-term monitoring to assess the success of control or eradication efforts.

Piaggio et al. [23] developed and successfully tested a conventional PCR primer set on a limited number of samples to detect Burmese python eDNA in south-eastern Florida. We chose to use qPCR technology for increased precision, sensitivity, specificity of detection, and reduction of false positives in eDNA samples [4,9,13,24–29]. Specifically, the highly sensitive TaqMan (Applied Biosystems, Foster City, CA) qPCR method uses three sequence-specific markers (two primers and a fluorescently-labelled probe) to detect the target DNA and can be used to quantify the number of copies or relative amounts of targeted molecules in the sample. Wilcox et al. [26] demonstrated that this method was able to detect a single copy of brook trout DNA in water samples.

In this study we had three objectives: (1) to develop and evaluate species-specific TaqMan qPCR primers and probes for five species of difficult to detect invasive giant constrictors (Burmese python, Northern African python, boa constrictor, and the green and yellow anaconda), (2) to use eDNA field-detections to geographically delineate presence of these constrictors, and (3) to calculate the first estimates of Burmese python occurrence using eDNA detected in water samples from various regions of southern Florida, while accounting for errors in detection using qPCR replicates.

## Methods

### Species-specific marker design

For Burmese python primer and probe designs, we aligned mitogenomic data from GenBank for the Burmese python, the closely related Indian Python (*P*. *m*. *molurus*), and Ball python (*P*. *regius*) to identify highly mutated species-specific regions in the NADH dehydrogenase subunit 4 (ND4). These regions along with ND4 gene sequences from Burmese pythons captured in Florida were used for assay design. Northern African python, boa constrictor, and green and yellow anaconda cytochrome *b* sequence data were aligned for the species of interest along with available data for related species. We aligned sequences in Geneious 5.4.7 or Sequencher 5.0, and loci were selected for qPCR TaqMan (Applied Biosystems, Foster City, CA) development. Primers and probes were designed for the Burmese python, Northern African python, boa constrictor, green anaconda, and yellow anaconda containing at between six and 14 sequence variants from related species in Primer Express Software version 3.0.1. Candidate primer sets were screened for positive amplification using conventional PCR techniques on one to 10 genomic DNA samples for each species [30]. The PCR products were purified using ExoSAP-IT for PCR clean-up (Affymetrix, Santa Clara, California, USA) and sequenced on an ABI 3730XL using the Big Dye Terminator v.3.1 kit (Applied Biosystems, Foster City, CA).

### Sample collection

#### Laboratory water trials

In the first phase of the study, field-captured or long-term captive snakes were submerged in tap water in sealable ActionPacker Storage containers (Rubbermaid). Containers for hatchlings measured 0.15 m^2^ by 0.35 m deep and those for adults were 0.3 m^2^ by 0.5 m deep. All containers were purchased new. Sterile technique and conditions were used during the laboratory trial. In each container, 7 L (hatchling) or 14 L (adults) of tap water were added 24 hrs prior to snake introduction. A negative control water sample was collected prior to each snake being introduced. One hatchling and two adult Burmese pythons, one adult boa constrictor, and one adult Northern African python were tested. Water samples were collected at 6 hrs, 24 hrs, 48 hrs, and 60 hrs after snake introduction to test for marker performance with increasing eDNA quantity. Samples were taken with the snake in the container. Room temperature was held between 24.8 and 25.7°C.

#### Field water samples

**Fig 1:**
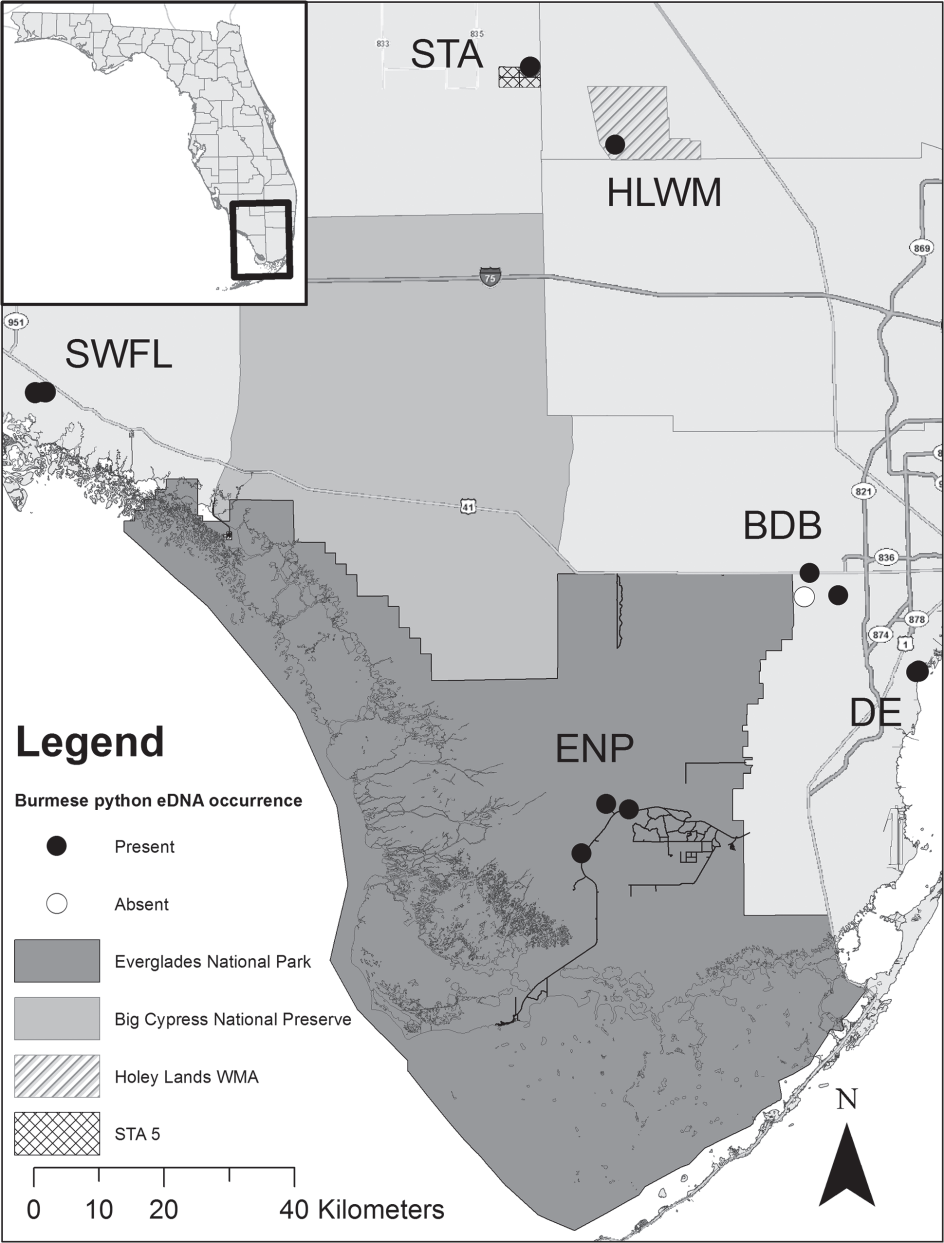
Map of study area with environmental DNA sample collection locations (N = 21) and Burmese python occupancy. Locations with present and absent environmental DNA detections are indicated with black and white dots, respectively. Some sample locations overlap due to the scale. BDB, Bird Drive Basin; DE, Deering Estates; ENP, Everglades National Park; HLWM, Holey Lands Wildlife Management Area; STA, Stormwater Treatment Area 5. Southwest Florida samples (SWFL) include three radiotagged Burmese pythons: ELV, Elvis; NOS, Noosa; and SWP, Sweet Pea.

In the second phase, water was collected and preserved in the field as described above during November 2013. Within each region, one to three locations were sampled in triplicate (field samples) resulting in three to nine samples per location (Fig 1). As proof of concept, field samples were collected within 60 m of three simultaneously tracked radiotelemetered adult Burmese pythons (“Sweet Pea” (SWP), “Noosa” (NOS), and “Elvis” (ELV)) from Collier County in southwest Florida. The three snakes were part of a separate study on movement and behavior. Noosa was found on a grassy basking platform above the water. Sweet Pea was tracked to a clump of vegetation in a grey water treatment pool, however no part of the snake’s body was visually identified. And Elvis was tracked, but not visually identified to a burrow 60 m from a pond where the samples were collected.

Next we sampled four regions without radiotelemetered snakes where constrictors have been sighted or captured in the past. Although experienced surveyors conducted the sample collection, no constrictor snakes were visually identified during the survey. We collected water samples at three locations in ENP with high Burmese python occupancy documented by multiple captures and previous radiotelemetric research. Within the region known to be occupied by Northern African pythons (Bird Drive Basin (BDB); Fig 1) on the western fringe of the city of Miami, water was collected near the locations of the three most recent captures of Northern African pythons. The Northern African python is considered to be established in this area, since over 25 snakes have been captured along with a small number of Burmese pythons (see Reed et al. [20]). Water samples from three locations were also collected in a forested hammock habitat at the Deering Estate (DE) in southern Miami, which is inhabited by a population of boa constrictors [19]. Live Burmese pythons have been captured less than five km from DE, but have not previously been documented there. Samples were collected from the nearest waterbody 500 m from a radiotelemetered boa constrictor that was in a dry upland burrow on our sampling date. Finally, Holey Land Wildlife Management Area (HLWM) bordered by Rotenberger and Everglades Wildlife Management Area, represents a northern extreme of the remaining Everglades sawgrass marsh and is managed by the State of Florida. Holey Land Wildlife Management Area was sampled at one location opportunistically one day after an eyewitness report of a large snake, putatively identified as an anaconda or Burmese python. Stormwater Treatment Area 5 (STA) was also opportunistically sampled at two locations one day after a Burmese python was sighted by an STA worker. These state lands contain canals and flooded wetlands suitable as habitat for giant constrictor snakes, although constrictors have not been sighted there previously.

### DNA extraction

#### Tissue sample DNA extraction

We isolated genomic DNA from Burmese python and Northern African python specimens collected from established populations in southern Florida, and from tissue samples and shed skins from Northern African python, boa constrictor, green anaconda, yellow anaconda, and the Ball python (a much smaller species closely related to the Northern African python which is common in the pet trade). Frozen muscle tissue and shed skin were isolated following Qiagen’s DNeasy Blood and Tissue kit (Valencia, CA) protocol. DNA quality was assessed using spectrophotometric absorbance and electrophoresis on a 1% agarose gel. Filter pipet tips were used throughout the study.

This study was carried out in strict accordance with the recommendations in the Guide for the Care and Use of Laboratory Animals of the National Institutes of Health and the Animal Welfare Act. The protocol was approved by the US Geological Survey, Southeast Ecological Science Center Institutional Animal Care and Use Committee (IACUC; Permit Number: USGS/SESC 2013–04). Additionally all samples were collected under the National Park Service (NPS; Everglades) Permit: EVER-2007-SCI-001 and EVER-2009-SCI-001. All efforts were made to minimize distress or suffering during handling. No constrictors were euthanized for the purposes of the study.

#### Water sample DNA extraction

We collected 950 mL of laboratory and field water samples in new 1 L DNase-free bottles (Nalgene) and preserved DNA with 1 mL of sodium acetate (3 M) and 33 mL of 95% ethanol. Samples were transported on dry ice for three to nine hrs and then frozen at -20°C until DNA extraction. After thawing, the sample was filtered through a sterile, disposable cellulose nitrate filter (0.45 μm Nalgene) using a vacuum pump. Filtering proceeded until the sample was completely processed or until water could not move through the filter. All filtration equipment was rinsed with distilled water between samples. Filters were frozen after filtration. DNA was extracted from frozen filters using the PowerWater DNA Isolation kit (MoBio Laboratories, Inc., Carlsbad, CA), which has been shown to perform well as compared to Phenol-Chloroform-Isoamyl alcohol and Qiagen DNeasy Blood and Tissue kit extractions [31]. Extracted DNA was suspended in 60μL buffer and quantified using an EPOCH spectrophotometer (BioTek, Winooski, VT). Samples with DNA yields less than 10 ng/μL were processed through a standard ethanol precipitation to increase DNA concentration [32]. Ethanol-precipitated pellets were re-suspended with 25 μL 10 mM Tris. Field negatives were prepared for each of the four targeted collection regions but not for the two opportunistic collection regions (HLWM and STA; Fig 1).

### Environmental DNA quantitative PCR

In order to obtain high levels of primer sensitivity and specificity of DNA from water samples, qPCR TaqMan assays were run with the developed primers and probes (Table 1) [13,26]. The probes were labeled with 6-FAM and MGB (5’and 3’, respectively). Following Applied Biosystems TaqMan protocols, the theoretical lower limit of final probe concentration was determined to allow for yields of the minimum C_t_ value for each target. Probe dilutions ranged from 50–250 nM in 50 nM increments. A TaqMan Exogenous Internal Positive Control (IPC; Applied Biosystems, Foster City, CA) was used to distinguish between true target negatives and PCR inhibition. Each 20.4 μL reaction contained a final concentration of 150nM of TaqMan probe in 1x PCR TaqMan Fast Advanced Master Mix (Applied Biosystems, Foster City, CA), 300 nM of each primer, 0.4 μL of Exo IPC DNA, and 2.0 μL Exo IPC Mix. An ABI StepOne-Plus Real-Time PCR system was used with the Fast qPCR profile consisting of 50°C (2 min) and 95°C (2 sec), followed by 40 cycles of 95°C (1 sec) and 20 sec at noted annealing temperatures (Table 1). Each set of qPCR markers was tested on between one (yellow anaconda) and 10 individuals of the target species to confirm amplification, in addition to on the other species described here and Ball pythons to confirm specificity. To identify theoretical thresholds of qPCR detection, 1:10 serial dilutions of DNA were conducted from 1 ng/μL to 1 X 10^–7^ ng/μL and were refined in 1:4 serial dilutions between the lowest and non-detected dilution. To verify that the primers did not cross-react with non-target species, we tested individuals from each species listed above for amplification at 1 X 10^–1^ ng/μL. We developed relative standard curves (RSC) from 1:10 serial dilutions of quantified target-species tissue DNA (1 ng/μL to 1 X 10^–7^ ng/μL; Table 2). The identified RSC slope and y-intercept were used to calculate the DNA concentration for the measured threshold cycle (C_t_) values.

**Table 1:** Environmental DNA quantitative PCR (qPCR) primer and probe sequences, annealing temperature (T_A_), and product size in base-pairs for five giant constrictor invasive species.

Species	Marker	Primer Forward (5’-3’)	Primer Reverse (5’-3’)	Probe (5’-3’)	T_A_	Product size (bps)
*Boa constrictor*	Bc-eDNA-cytb	CAT GGT GAA ACT TCG GAT CAA TAC	TGT GTA ATG TAC GGC CAA GAA GA	ATG CTC CAT AAT TCA AGT AC	57	83
*Eunectes murinus*	En-eDNA-cytb	TCG GCC TAT TAC CAG TAG CAA	TGC TAG GTT GAT GTT TGC TGT	TGC TGC TTG CCT GCT CAA CCC T	63	134
*Eunectes notaeus*	Em-eDNA-cytb	GGT GGA GCA CTA GCC CTA ACA A	TGC TGC GAA CAG TCA GAA TGT	CGT AAT TCT CCT CAC AGT C	60	126
*Python molurus bivittatus*	Pmb-eDNA-ND4	CAC CCTA ACA ACT TCA ATA CCT CTA CTA AT	GAG GTT TGT TCA GTG GTT ATT TGT TTT	CCA ACA CTA TTA TTC CTA GCA AC	60	146
*Python sebae*	Ps-eDNA-cytb	AAT CAC CAA CCT ACT CAC TGC TGT AC	GCC TCC CCA TAA TCA GGT TGT	CTA CCT AGG AAC AAC TCT	63	67

**Table 2:** Relative quantification (relative standard curve) values for five giant constrictor species using genomic DNA (gDNA) and absolute quantification (standard curve) values for *Python molurus bivittatus* using a synthetic gene.

Quantification type	Species	Marker	Slope	R^2^	Efficiency (%)	Y-intercept
Relative standard curve (gDNA)						
	*Boa constrictor*	Bc-eDNA-cytb	-3.57	0.997	90.68	25.83
	*Eunectes notaeus*	En-eDNA-cytb	-3.44	0.998	95.44	23.39
	*Eunectes murinus*	Em-eDNA-cytb	-3.46	0.996	94.61	29.27
	*Python molurus bivittatus*	Pmb-eDNA-ND4	-3.13	0.997	108.05	21.14
	*Python sebae*	Ps-eDNA-cytb	-3.51	0.993	92.85	21.87
Standard curve (synthetic gene)						
	*Python molurus bivittatus*	Pmb-eDNA-ND4	-3.51	0.995	92.72	7.97

A slope of −3.1 to −3.6, R^2^ > 0.99, and 90–110% PCR efficiency are required for accurate target quantification.

**Fig 2:**
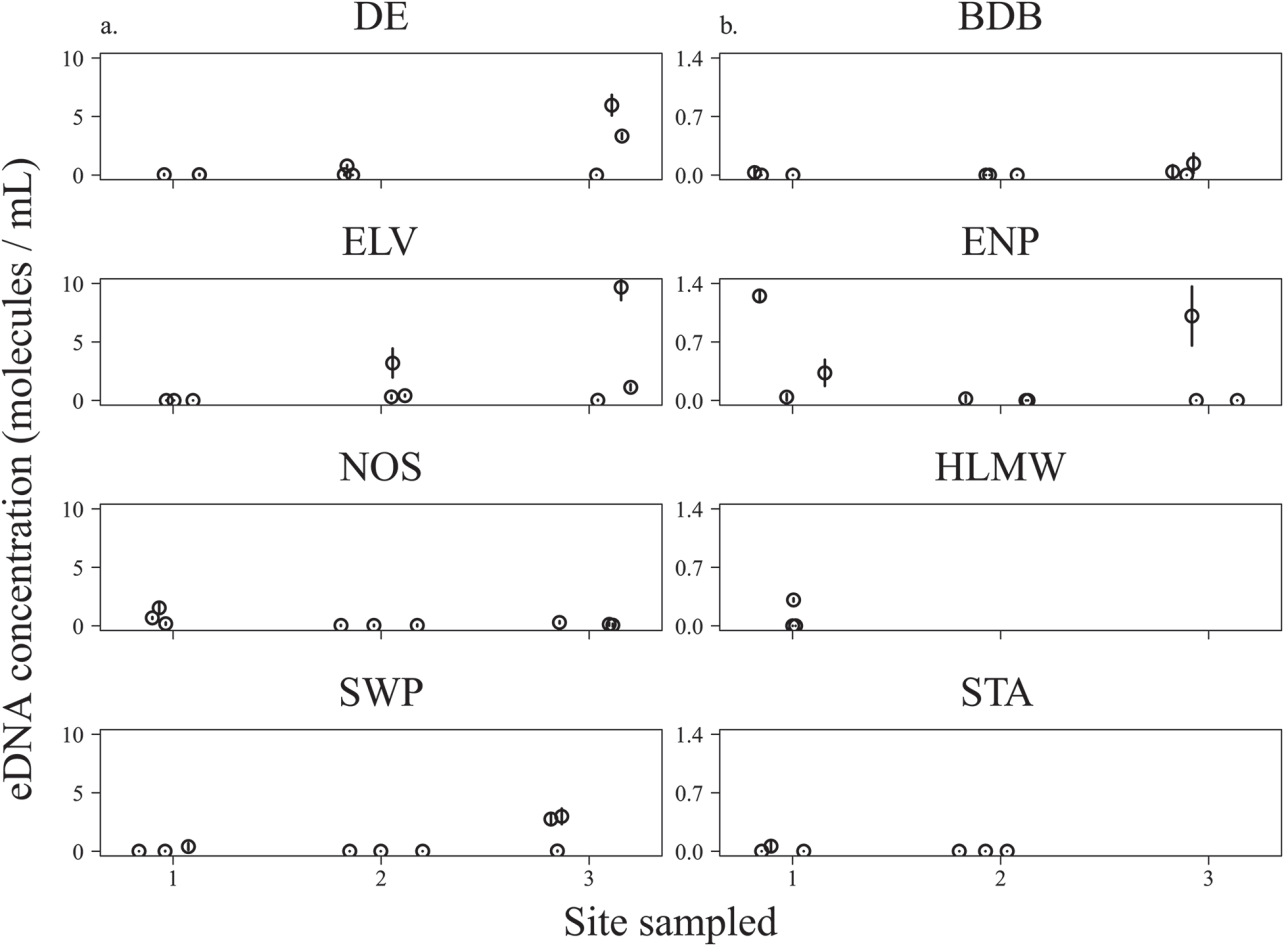
Estimated concentration of Burmese python environmental DNA (mean and 95% confidence interval) per sample. a. Higher concentration regions, y-axis scaled from 0 to 10 molecules/mL. b. Lower concentration regions, y-axis scaled from 0.0 to 1.4 molecules/mL. From each region, one to three locations were targeted, with triplicate field samples collected at each location. BDB, Bird Drive Basin; DE, Deering Estates; ENP, Everglades National Park; HLWM, Holey Lands Wildlife Management Area; SWP, Sweet Pea; NOS, Noosa; ELV, Elvis; STA, Stormwater Treatment Area 5.

Eight regions throughout southern Florida were assessed at one, two, or three locations with triplicate field samples per location resulting in 63 field samples from 21 locations. Water was collected from approximately 10 cm below the surface. Eluted samples were analyzed in triplicate qPCRs (technical replicates), resulting in up to 27 qPCRs per location, respectively. Negative (sterile water) and positive controls (DNA isolated from tissue samples) were included in each qPCR and a negative control of sterile water was included for each laboratory trial and field region to detect any cross-contamination of reagents or equipment. The average sample DNA concentration for the three technical replicates was calculated (Fig 2, S1 Table). The DNA concentration was computed based on the filtered volume. Technical replicate eDNA concentrations below the theoretical detection limit were excluded from further analysis (see results). Positive samples were sequenced using conventional PCR for species confirmation. The Burmese python assay was screened on all 63 field samples. The Northern African python assay was tested on BDB and DE samples, the boa constrictor assay was tested on DE, and the green and yellow anaconda assays were tested on HLWM samples.

### Quantitative PCR copy number quantification

Since only Burmese pythons were detected from field water samples, we calculated initial copy number of eDNA molecules using a synthetic gene. Concentrations based on relative standard curves are provided for the four other species. To quantify the copy number of Burmese python eDNA samples, a 146 bp custom gene was synthesized and inserted into a vector for replication (Integrated DNA technologies, IDT, Coralville, Iowa). The desiccated synthetic gene was re-suspended in 50 μL and linearized using ApaI restriction enzyme (New England BioLabs, Ipswich, MA). The product was purified using an Agarose Gelextract Mini kit and eluted with 50μL of PEB buffer (5 Prime Inc., Gaithersburg, MD). The pure fragment was quantified on a Qubit 2.0 fluorometer (Invitrogen) and diluted to a final concentration of 10^5^ molecules/μL. We then used serial dilutions to create a standard curve for determining the copy number of double-stranded DNA (dsDNA) targets in eDNA samples. All test and experimental samples were analysed in triplicate qPCRs.

### Probability of occurrence and detection

For each of the regions surveyed in southern Florida, we used a multi-scale occupancy model to estimate probabilities of occurrence and detection of python eDNA. Our modelling approach was similar to that described by Schmidt et al. [10], except that the effects of covariates were not included. Specifically, let *y*_*ij*_ ∈ {0,1,…,*K*} denote the number of technical replicates wherein python eDNA was detected from the *j*th water sample collected at survey location *i*(*i* = 1,…, *m*; *j* = 1,…, *J*_*i*_). We used the qPCR assay's limit of detection as a threshold for computing *y*_*ij*_ from our measurements of python eDNA concentration. To analyze these data, we assumed a hierarchical occupancy model with the following three levels:

$$\begin{array}{c} Y_{ij}|A_{ij}=a_{ij}∼\text{Binomial }\left(K,\;a_{ij}p\right)\\ A_{ij}|Z_{i}=z_{i}∼\text{Bernoulli }\left(z_{i}\theta_{i}\right)\\ Z_{i}∼\text{Bernoulli}\;\left(\psi \right) \end{array}$$

where *A*_*ij*_ is a latent (unobserved) binary random variable for the occurrence (i.e., presence (*A*_*ij*_ = 1) or absence (*A*_*ij*_ = 0)) of python eDNA in the *j*th sample of location *i*. Similarly, *Z*_*i*_ denotes a latent binary random variable for the occurrence of python eDNA at the *i*th survey location.

The focus of our analysis was to estimate the parameters of this model for each of the regions surveyed in southern Florida. The parameters of interest included *ψ* (probability of occurrence of python eDNA among all surveyed locations), *θ*_*i*_ (conditional probability of occurrence of python eDNA in each sample of location *i*, given that python eDNA was present at that location), and *p* (conditional probability of detection of python eDNA in each qPCR replicate of an eDNA sample, given that python eDNA was present in the sample).

In addition to these parameters, we were interested in computing inferences for two derived parameters of the model. The first of these parameters was p* = 1−(1−*p*)^*K*^, which denotes the cumulative probability of detecting python eDNA in *K* qPCR replicates of an eDNA sample, given that the sample contained python eDNA. Estimating p* allowed us to assess whether the *K* = 3 technical replicates per sample had been sufficient to detect python eDNA in samples that contained python eDNA. The second derived parameter of interest was$\theta^{*}\;=\;1-{\left(1-\bar{\theta }\right)}^{n}$, which denotes the cumulative probability of occurrence of python eDNA in *n* samples taken from a location that contained python eDNA. We computed θ* for a sequence sample sizes (*n* = 1,2…) using an estimate of$\bar{\theta }=\left(\overset{m}{\underset{i=1}{\sum }}z_{i}\theta_{i}\right)/\overset{m}{\underset{i=1}{\sum }}z_{i}$), the average conditional probability of occurrence of python eDNA in a single sample averaged over all survey locations where python eDNA was actually present. Estimating θ* allowed us to assess whether the number of eDNA samples collected per survey location had been sufficient to include python eDNA when python eDNA was present at that location.

We fitted the multi-scale occupancy model to python eDNA data using Bayesian methods of analysis. The technical details of the analysis (prior distributions of model parameters, Markov chain Monte Carlo algorithm, and assessment of Monte Carlo error) and the source code are provided in S1 and S2 Appendix. The analysis was conducted using the R software program [33].

## Results

### Species-specific PCR marker design

From aligned sequences, primers and probes were designed for the Burmese python using the ND4 region (Table 1). Northern African python, boa constrictor, green anaconda, and yellow anaconda primers and probes were developed using cytochrome *b* (Table 1). No cross-species amplification was identified. Using serial dilutions of known amounts of DNA, the theoretical lower limits of detection for the Burmese python had a RSC threshold at 8 X 10^−6^ ng/μL (C_t_ = 36.3–38.6) and a synthetic gene standard curve threshold of 4 x 10^−10^ ng/μL (C_t_ = 36.5–38.4) or 0.363 average number of molecules in a PCR assay. The standard curve based on the synthetic gene was suitable to estimate copy numbers of unknown samples (Table 2). The relative standard curve concentrations are also provided for comparison with the four other species. The theoretical lower limit of detection for the Northern African python was 1 X 10^−4^ ng/μL (C_t_ = 36.69–36.97). The theoretical lower limit of detection for the boa constrictor was 1 X 10^−3^ ng/μL (C_t_ = 36.16–36.98). And the theoretical lower limit of detection for the green anaconda was 6 X 10^−3^ ng/μL (C_t_ = 36.47–39.27) and yellow anaconda at 1 X 10^−4^ ng/μL (C_t_ = 36.83–37.9).

### Laboratory water trials

DNA was extracted from 25 laboratory water samples and resulted in concentrations ranging from 3.5 to 311.0 ng/μL. All laboratory trials were positive for the species tested, resulting in a detection probability of one (S1 Fig). All negative control samples resulted in no amplification of DNA. A positive correlation between time and DNA concentration was found, although the relationship was not linear. This may have been due to uneven mixing of the water prior to sampling or snake behavior, including variations in activity and defecation, or even snakes climbing onto the sides of the containers and out of the water (S1 Fig).

### Field water trials

In each of 21 locations throughout southern Florida (Fig 1), three samples along with four negative field controls were collected for a total of 67 field samples. The concentration of DNA from environmental water ranged from 0.0 to 309.5 ng/μL. Quantitative PCR assay controls provided expected positive and negative results for each assay. All IPC assays on field water samples indicated appropriate qPCR curves and no inhibition. DNA from the Northern African python, boa constrictor, green anaconda, and yellow anaconda was not detected in any tested field samples. However, Burmese python DNA was detected within all eight regions in 17 locations and in 37 field samples (S1 Table). Of the positive eluted samples, a subset of 24 field samples was sequenced and all provided accurate Burmese python DNA sequences.

Burmese python eluted DNA concentrations (averaged over technical replicates) ranged from 0.35–161.24 molecules/μL (ENP2C and ELV3C, respectively; S1 Table). Deering Estate and ENP samples resulted in a wide range of concentrations and seven and five positive locations out of nine, respectively. Bird Drive Basin had only three positive locations. Of the opportunistically collected samples, one water sample was positive in both HLWM and STA.

The samples collected near radio-tagged snakes contained higher average levels of eDNA (14.38 molecules/μL) as compared to samples without a radiotracked snake in the region (6.74 molecules/μL). Assays from the three tracked wild Burmese pythons resulted in 58.02% positive technical replicates (S1 Table). The sampling events ELV2, NOS1, 2, 3 and SWP3 were positive for the triplicate field samples. Over all samples, ELV3C had the highest technical replicate concentration (177.71 molecules/μL) and NOS had the most positive technical replicate assays (74.07%).

### Probability of occurrence and detection

We were able to fit the multi-scale occupancy model to seven of the eight regions that were surveyed for python eDNA. The model could not be fitted to HLWM because eDNA samples were collected from only one location in that region. Estimates of detection probabilities of python eDNA suggested that qPCR was effective in detecting eDNA presence in a sample (Table 3, S2 Table). For example, estimated detection probabilities ranged from 0.59 to 0.87. In addition, estimates of the cumulative probability of detecting python eDNA (p*) ranged from 0.91 to 1.00, suggesting that three qPCR replicates per eDNA sample were sufficient to detect python eDNA when it was present in a sample.

**Table 3:** Regional Bayesian estimates of occurrence (*ψ*) and detection (*p*) probabilities of *Python molurus bivittatus* environmental DNA.

Region	*ψ*	95% CI	$\bar{\theta }$95% CI	*p*	95% CI	p*	95% CI
BDB	0.67	0.22–0.98	0.51	0.23–0.82	0.59	0.31–0.85	0.91	0.67–1.00
DE	0.80	0.40–0.99	0.69	0.46–0.89	0.64	0.44–0.81	0.94	0.82–0.99
ENP	0.80	0.40–0.99	0.54	0.32–0.75	0.80	0.59–0.94	0.99	0.93–1.00
STA	0.57	0.11–0.97	0.39	0.07–0.83	0.73	0.35–0.96	0.95	0.73–1.00
ELV	0.69	0.23–0.99	0.72	0.44–0.96	0.87	0.71–0.97	1.00	0.98–1.00
NOS	0.80	0.40–0.99	0.80	0.59–0.95	0.75	0.59–0.88	0.98	0.93–1.00
SWP	0.64	0.21–0.97	0.68	0.39–0.92	0.74	0.53–0.91	0.98	0.89–1.00

Posterior mean and 95% credible interval (CI) are given for each parameter of the occupancy model. Average conditional probability of occurrence of Burmese python eDNA in a single sample ($\bar{\theta }$). Cumulative probability of detecting eDNA in three quantitative PCR replicates (p*). BDB, Bird Drive Basin; DE, Deering Estates; ENP, Everglades National Park; HLWM, Holey Lands Wildlife Management Area; STA, Stormwater Treatment Area 5; and radiotagged snakes: ELV, Elvis; NOS, Noosa; and SWP, Sweet Pea.

**Fig 3:**
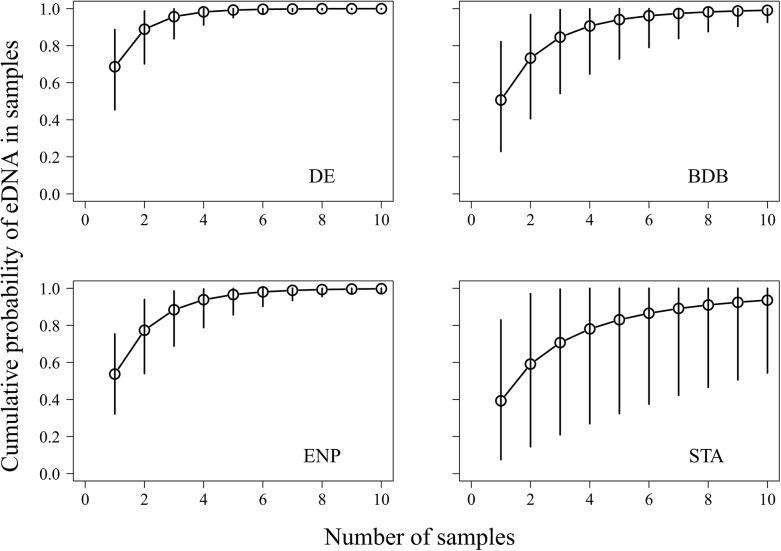
The effect of sample size on estimates of the cumulative probability of occurrence of Burmese python environmental DNA (θ*) associated with samples taken from the four field locations analyzed by the three-level occupancy model. BDB, Bird Drive Basin; DE, Deering Estates; ENP, Everglades National Park; STA, Stormwater Treatment Area 5.

Estimated probabilities of occurrence of python eDNA ranged from 0.57 to 0.80 (Table 3). Our estimates of $\bar{\theta }$ ranged from 0.39 to 0.80, suggesting that python eDNA was not present in every sample collected from survey locations where python eDNA was present. However, estimates of the cumulative probability of eDNA occurrence (θ*) suggested that python eDNA was highly likely to be present in at least one of the three samples collected from each survey location, provided, of course, that python eDNA was present at the location (Fig 3). In ENP, for example, the cumulative probability that python eDNA was present in at least one of three samples was 0.88, though considerable uncertainty was present in our estimate of this parameter (95% credible interval for θ* = 0.69–0.98).

## Discussion

### Probability of occurrence and detection of invasive Burmese pythons

The distribution and abundance of Burmese pythons in southern Florida are not known with any degree of certainty. Motivated by this dearth of knowledge, we collected samples of python eDNA using a generic design which included three stages: (1) selection of survey locations from each region of interest, (2) collection of multiple eDNA samples within each of these locations, and (3) analysis of qPCR replicates (subsamples) taken from each sample of python eDNA.

While our samples of southern Florida were limited in number, they did reveal considerable heterogeneity in the average concentration of eDNA among regions, among locations within regions, and even among samples within locations (Fig 2, S1 Table). In contrast, the eDNA concentrations of qPCR replicates taken from the same sample were usually quite similar (S1 Table), suggesting that the differences of eDNA concentration among samples were associated with differences in the occurrence or abundance of pythons as opposed to differences in our ability to detect python eDNA.

Multi-scale occupancy modeling allowed us to estimate probabilities of occurrence of python eDNA (for locations or samples) while also estimating the conditional probability of detecting python eDNA that may have been present in a sample. In fact, our occupancy-based analysis of python eDNA provided the first estimates of Burmese python detection probabilities that were substantially higher than zero, signaling a dramatic improvement over previous attempts to detect pythons in the GEE [22]. Our estimated probabilities of detection suggested that Burmese python eDNA can be detected quite reliably (when present) in 1 L samples and that false negatives were of limited concern. In general, increasing the volume of the sample would improve the ability to detect eDNA, while increasing the number of technical replicates could increase the precision of the estimated detection probability.

These results are similar to those of Wilcox et al. [26], who reported perfect detection in high numbers of qPCR replicates taken from samples of trout eDNA containing concentrations ranging from 2.5 to 312.5 molecules/μL. At the lowest concentration (0.5 molecules/μL), Wilcox et al. [26] reported detection probabilities ranging from 0.57–0.74; therefore, as in our study, only a few qPCR replicates were needed to conclude with certainty whether eDNA was present or absent in a sample. At the locations we surveyed, three eDNA samples appeared to be adequate for obtaining accurate estimates of python eDNA occurrence (Fig 3); however, we recommend that future surveys of python eDNA include more locations (e.g., ten or more) to estimate the occurrence of Burmese pythons more accurately in the GEE.

The multi-scale (three-level) occupancy model described by Schmidt et al. [10] can be used to standardize the terminology used in eDNA-based surveys and to eliminate confusion between occurrence and detection. This model is consistent with the generic eDNA sampling design that we advocate wherein (1) multiple locations are selected to be representative of some region of interest, (2) multiple eDNA samples are collected at each location, and (3) each eDNA sample is subsampled to obtain quantitative PCR (qPCR) replicate observations of eDNA (that is, detections and/or nondetections). Using this design, detections of eDNA occur at the level of individual qPCR replicates. If eDNA is present in a sample, a conditional probability of detecting eDNA in each qPCR replicate is well defined and estimable using occupancy models. Similarly, if eDNA is present at a survey location, the conditional probability of eDNA occurring in a sample is well defined and estimable using occupancy models. Environmental DNA can be absent or present in samples collected from a location where the target species is present [14]. For example, in the analysis of Schmidt et al. [10], eDNA was present in only 45% of the samples collected from sites where eDNA of the target species was known to occur.

### Factors in eDNA detection of invasive constrictors in southern Florida

The highest concentrations of Burmese python eDNA were identified in DE and around the three radiotelemetered constrictors, although these regions also returned some negative samples, highlighting eDNA heterogeneity (Fig 2) [14]. Sweet Pea was found in a wastewater treatment pool where chemical degradation of DNA could have been a factor and NOS was found on a dry grassy basking platform above the water, possibly reducing the suspended eDNA. The highest sample and location concentrations were found in a pond 60 m from ELV, possibly suggesting the presence of undetected pythons. Everglades National Park, DE, and ELV had the largest variation among samples and locations.

Burmese pythons eDNA detection in DE may indicate presence at low densities, or the detected eDNA may have come into the region through canals or drainage flow from areas to the west that are unequivocally occupied. We did not detect boa constrictor DNA in water samples collected at DE, possibly due to their largely terrestrial habits. Burmese pythons also were detected in water samples collected from BDB; however the Northern African python was not detected, possibly due to low densities or reduced use of water habitat. Two eDNA detections were from the leading northern edge of the invasive Burmese python population (STA and HLWM). Since the sheet flow in the GEE moves from the north to the south, the reported concentrations are likely conservative. Although almost entirely aquatic, we also did not detect the green or yellow anaconda in any field samples, indicating low densities or limited ranges for the species. Additional water samples per location or more spatially intensive sampling may be needed to achieve positive detections.

Low eDNA concentrations or a lack of positive detections in locations suspected of containing snakes may be due to environmental conditions [34]. Season, water flow, temperature, and other environmental variables may affect the retention and stability of DNA in water. Alternatively, variations in sampling location, density of the species in that region, environmental heterogeneity and assay sensitivity and detection threshold could affect positive detections. Based on our results and other published studies, variation among samples (e.g. environmental heterogeneity) within the same location and/or region is common [14].

Environmental DNA abundance has been positively correlated with population density and biomass [4,6,9,10,27,34]. Although giant constrictors are large, travel widely, and eat frequently, physiological factors may limit detection of their DNA in water samples. Compared to fish, semi-aquatic snakes typically spend less time in the water, defecate less frequently, and have lower activity levels. Another factor that may limit cell turnover is the lack of continual shedding of the outermost layer of reptile skin. Among pythons in Florida, shedding tends to occur on land. In comparison, the high turnover rate of mucus layers and scales of fully-aquatic organisms provides a steady source of cellular material for eDNA detection [4,9].

### Future directions and conclusions

The sensitive and specific eDNA tools presented here can improve estimates of detection probabilities for cryptic and elusive giant constrictors. These methods can assist with early detection of non-established species and range delimitation for established species, especially as they move northward [35,36]. Further, to detect constrictors on land, these tools could be used with soil samples from game trails, burrow entrances, and levees to help delineate range limits.

These tools can be used to improve detection of pythons in occupied areas. However, as with other sampling strategies, these tools may not be informative if target species occur at very low densities or are non-uniformly distributed. Further, while eDNA is a promising method for determining detection and occupancy probabilities of invasive giant constrictors in an area, translating eDNA detections into snake captures may be difficult. Once an area is targeted for snake removal, individual snake detection strategies, such as detector dogs, may improve population control efforts.

Information on density effects, minimum volume, and number of PCRs is being further addressed using site occupancy models. Environmental DNA methodologies may also prove useful in determining critical habitats for conservation in Southern and Southeast Asia, where Burmese pythons are considered vulnerable by the International Union for the Conservation of Nature (IUCN) [37]. Future uses of these tools include assisting management efforts to limit colonization, assessing risk to native species, and informing the control efforts of constrictor populations.

## Supporting Information

S1 Appendix MCMC algorithm used to fit occupancy model. (PDF)

S2 Appendix Environmental DNA occupancy model source code for R package. (R)

S1 Fig Environmental DNA concentration over time during laboratory trials. Constrictor snakes were held in containers with 7 L (hatchling) or 14 L (adults) of water, which was sampled 4 times over 60hrs. Trial 1. Hatchling Burmese python; Trial 2. Adult Burmese python; Trial 3. Adult Burmese python; Trial 4. Adult Northern African python; and Trial 5. Adult boa constrictor.
(TIF)

S1 Table Burmese python *e*nvironmental DNA (eDNA) quantitative PCR technical replicate concentrations. Quantitative PCR replicate concentrations (molecules/μL) of Burmese python eDNA. From each region, one to three locations were targeted, with three sample replicates (A,B,C) collected at each location. The filtration (mL) and DNA extraction elution volumes (μL) are also provided. BDB, Bird Drive Basin; DE, Deering Estates; ENP, Everglades National Park; HLWM, Holey Lands Wildlife Management Area; SWP, Sweet Pea; NOS, Noosa; ELV, Elvis; STA, Stormwater Treatment Area 5.
(PDF)

S2 TableRegional Bayesian estimates of occurrence (*ψ*) and detection (*p*) probabilities of *Python molurus bivittatus* environmental DNA.Posterior mean and 95% credible interval (CI) are given for each parameter of the occupancy model. A uniform prior distribution was assumed. Average conditional probability of occurrence of Burmese python eDNA in a single sample ($\bar{\theta }$). Cumulative probability of detecting eDNA in three qPCR replicates (p*). BDB, Bird Drive Basin; DE, Deering Estates; ENP, Everglades National Park; HLWM, Holey Lands Wildlife Management Area; STA, Stormwater Treatment Area 5; and radiotagged snakes: ELV, Elvis; NOS, Noosa; and SWP, Sweet Pea.(PDF)
