# Supplementary material for: Environmental DNA (eDNA) Sampling Improves Occurrence and Detection Estimates of Invasive Burmese Pythons
Source: PLoS One. 2015 Apr 15;10(4):e0121655. doi: 10.1371/journal.pone.0121655 (PMC4398459; doi:10.1371/journal.pone.0121655)
Supplement: S1 Appendix — (PDF) [file pone.0121655.s001.pdf]

Appendix S1

MCMC algorithm used to fit occupancy model

In this supplement we describe the MCMC algorithm that was used to compute posterior summaries of the occupancy model's parameters. For this model the unnormalized posterior density function[1] is

$$[\psi, p, \boldsymbol{\theta}, \boldsymbol{z}, \boldsymbol{a}|\boldsymbol{y}] \propto [\psi, p, \boldsymbol{\theta}] \prod_{i=1}^{m} \psi^{z_i}(1-\psi)^{1-z_i} \prod_{j=1}^{J_i} (z_i\theta_i)^{a_{ij}}(1-z_i\theta_i)^{1-a_{ij}} \binom{K}{y_{ij}} (a_{ij}p)^{y_{ij}}(1-a_{ij}p)^{K-y_{ij}}$$

where $\boldsymbol{z} = (z_1, \ldots, z_m)'$, $\boldsymbol{\theta} = (\theta_1, \ldots, \theta_m)'$, and $\boldsymbol{a} = \{a_{ij}\}$. The term $[\psi, p, \boldsymbol{\theta}] = [\psi][p][\boldsymbol{\theta}]$ denotes the prior density. The MCMC algorithm that we used is a straightforward application of Gibbs sampling (Geyer, 2011). Each of following full-conditional distributions was sampled in one iteration of the algorithm:

1. The full conditional density of $\boldsymbol{z}$ factors into a product of m independent terms. The full conditional distribution of Z_i is:

$$Z_i|\cdot \sim \begin{cases} \text{Bernoulli}(1) & \text{if } \sum_{j=1}^{J_i} a_{ij} > 0 \\ \text{Bernoulli}\left(\frac{\psi(1-\theta_i)^{J_i}}{\psi(1-\theta_i)^{J_i}+1-\psi}\right) & \text{if } \sum_{j=1}^{J_i} a_{ij} = 0 \end{cases}$$

2. Assuming a Beta(a, b) prior distribution for ψ yields a full conditional distribution of familiar form: $\psi|\cdot \sim \text{Beta}(a + m\bar{z},\ b + m(1 - \bar{z}))$, where $\bar{z} = (1/m)\sum_{i=1}^{m} z_i$. Hyperparameters a and b were each assigned a value of one to specify a uniform prior distribution.

3. The full conditional density of $\boldsymbol{a}$ factors into a product of $\sum_{i=1}^{m} J_i$ independent terms. The full conditional distribution of A_{ij} is:

$$A_{ij}|\cdot \sim \begin{cases} \text{Bernoulli}(1) & \text{if } y_{ij} > 0 \\ \text{Bernoulli}\left(\frac{z_i\theta_i(1-p)^K}{z_i\theta_i(1-p)^K+1-z_i\theta_i}\right) & \text{if } y_{ij} = 0 \end{cases}$$

4. The full conditional density of $\boldsymbol{\theta}$ factors into a product of m independent terms. Assuming a Beta(e, f) prior distribution for each element of $\boldsymbol{\theta}$ yields full conditional distributions of familiar form: $\theta_i|\cdot \sim \text{Beta}(e + z_i\sum_{j=1}^{J_i} a_{ij},\ f + z_i(J_i - \sum_{j=1}^{J_i} a_{ij}))$. Hyperparameters e and f were each assigned a value of one to specify a uniform prior distribution for each element of $\boldsymbol{\theta}$.

5. Assuming a Beta(c, d) prior distribution for p yields a full conditional distribution of familiar form: $p|\cdot \sim \text{Beta}(c + \sum_{i=1}^{m}\sum_{j=1}^{J_i} a_{ij}y_{ij},\ d + \sum_{i=1}^{m}\sum_{j=1}^{J_i} a_{ij}(K - y_{ij})$. Hyperparameters c and d were assigned values $(c = 4, d = 1)$ to specify an informative prior whose density increases monotonically with p and peaks at $p = 1$ (i.e., the prior's

[1]We use bracket notation (Gelfand and Smith, 1990) to specify probability density functions; thus, $[x, y]$ denotes the joint density of random variables X and Y, $[x|y]$ denotes the conditional density of X given $Y = y$, and $[x]$ denotes the unconditional (marginal) density of X.

mode is finite). We selected this prior because the probability of detecting eDNA in a sample that contained eDNA was expected to have been high given the sensitivity of qPCR-based measurements of eDNA. We investigated the influence of this prior on our results by also fitting an occupancy model that assumed a uniform prior distribution for p (i.e., $c = 1, d = 1$). Assuming a uniform prior for p had little effect on estimates of ψ and $\boldsymbol{\theta}$ (cf. Table 3 and Table S2 (Supporting Information)), but the uniform prior did result in somewhat lower estimates of p as might be expected given the low number of qPCR replicates ($K = 3$) per sample of eDNA.

We used $M = 100000$ iterations of the MCMC algorithm to estimate summaries (means, standard deviations, quantiles) of the posterior distribution and other relevant functionals of the Markov chain. The estimates were computed using ergodic averages, which are simulation consistent (that is, the averages converge to posterior expectations as the number of iterations increases) according to the strong law of large numbers for Markov chains (Flegal and Jones, 2011). (The first 5000 elements of the Markov chain were discarded as potentially transient parts of the chain.) Monte Carlo standard errors of the estimates were computed using the subsampling bootstrap method (Flegal and Jones, 2010, 2011) with overlapping batch means of size $\lfloor\sqrt{M}\rfloor$. The Monte Carlo error of any posterior summary was never higher than 0.002 given our choice of M; thus, we used two significant digits to report a reliable estimate of each posterior summary.

References

Flegal, J. M. and Jones, G. L. (2010). Batch means and spectral variance estimators in Markov chain Monte Carlo. *Annals of Statistics*, 38:1034–1070.

Flegal, J. M. and Jones, G. L. (2011). Implementing MCMC: estimating with confidence. In Brooks, S., Gelman, A., Jones, G. L., and Meng, X. L., editors, *Handbook of Markov chain Monte Carlo*, pages 175–197. Chapman & Hall / CRC, Boca Raton, Florida.

Gelfand, A. E. and Smith, A. F. M. (1990). Sampling-based approaches to calculating marginal densities. *Journal of the American Statistical Association*, 85:398–409.

Geyer, C. J. (2011). Introduction to Markov chain Monte Carlo. In Brooks, S., Gelman, A., Jones, G. L., and Meng, X. L., editors, *Handbook of Markov chain Monte Carlo*, pages 3–48. Chapman & Hall / CRC, Boca Raton, Florida.
