# Supplementary figures and images for: Environmental DNA (eDNA) Sampling Improves Occurrence and Detection Estimates of Invasive Burmese Pythons
Source: PLoS One. 2015 Apr 15;10(4):e0121655. doi: 10.1371/journal.pone.0121655 (PMC4398459; doi:10.1371/journal.pone.0121655)

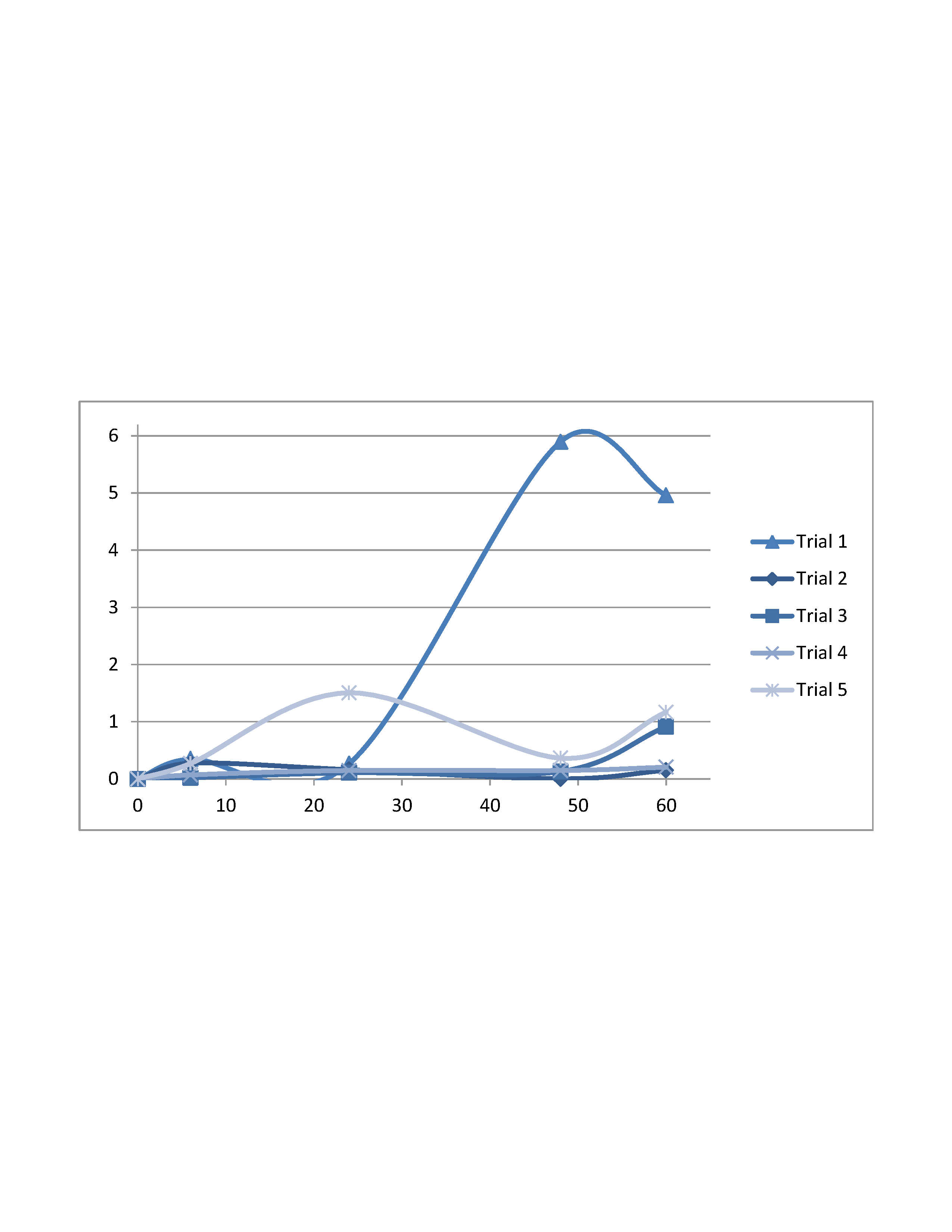

Supplement: S1 Fig — Constrictor snakes were held in containers with 7 L (hatchling) or 14 L (adults) of water, which was sampled 4 times over 60hrs. Trial 1. Hatchling Burmese python; Trial 2. Adult Burmese python; Trial 3. Adult Burmese python; Trial 4. Adult Northern African python; and Trial 5. Adult boa constrictor. (TIF) [file pone.0121655.s003.tif]
