# Supplementary material for: Environmental DNA (eDNA) Sampling Improves Occurrence and Detection Estimates of Invasive Burmese Pythons
Source: PLoS One. 2015 Apr 15;10(4):e0121655. doi: 10.1371/journal.pone.0121655 (PMC4398459; doi:10.1371/journal.pone.0121655)
Supplement: S1 Table — Quantitative PCR replicate concentrations (molecules/μL) of Burmese python eDNA. From each region, one to three locations were targeted, with three sample replicates (A,B,C) collected at each location. The filtration (mL) and DNA extraction elution volumes (μL) are also provided. BDB, Bird Drive Basin; DE, Deering Estates; ENP, Everglades National Park; HLWM, Holey Lands Wildlife Management Area; SWP, Sweet Pea; NOS, Noosa; ELV, Elvis; STA, Stormwater Treatment Area 5. (PDF) [file pone.0121655.s004.pdf]

Supporting Information Table S1. Burmese python environmental DNA (eDNA) quantitative PCR technical replicate concentrations. PCR replicate concentrations (molecules/μL) of Burmese python eDNA. From each region, one to three locations were targeted, with three sample replicates (A,B,C) collected at each location. The filtration (mL) and DNA extraction elution volumes (μL) are also provided. BDB, Bird Drive Basin; DE, Deering Estates; ENP, Everglades National Park; HLWM, Holey Lands Wildlife Management Area; SWP, Sweet Pea; NOS, Noosa; ELV, Elvis; STA, Stormwater Treatment Area 5.

Sample	Replicate 1	Replicate 2	Replicate 3	Filtered volume (mL)	Eluted volume (uL)
BDB1A	0.00	0.00	0.00	650	60
BDB1B	0.00	0.00	0.00	500	25
BDB1C	1.67	0.00	0.00	500	25
BDB2A	0.00	0.00	0.00	1000	60
BDB2B	0.00	0.00	0.00	1000	60
BDB2C	0.00	0.00	0.00	1000	60
BDB3A	2.22	0.00	0.00	1000	60
BDB3B	1.33	1.26	4.19	1000	60
BDB3C	0.00	0.00	0.00	1000	60
DE1A	1.73	0.00	0.00	1000	60
DE1B	1.28	0.00	0.00	1000	60
DE1C	1.50	0.00	0.00	1000	60
DE2A	0.00	0.00	0.00	1000	60
DE2B	1.68	0.00	0.00	1000	60
DE2C	12.83	11.97	14.27	1000	60
DE3A	0.00	0.00	0.00	1000	60
DE3B	125.23	101.74	131.23	500	25
DE3C	59.82	53.46	52.98	1000	60
ENP1A	25.94	24.99	24.01	500	25
ENP1B	9.73	5.15	4.85	500	25
ENP1C	1.47	1.08	0.00	500	25
ENP2A	0.00	0.00	0.00	500	25
ENP2B	0.00	0.00	0.00	500	25
ENP2C	1.05	0.00	0.00	500	25
ENP3A	0.00	0.00	0.00	500	25
ENP3B	0.00	0.00	0.00	1000	25
ENP3C	13.36	9.79	7.14	250	25
HLWM1A	0.00	0.00	0.00	1000	60
HLWM1B	0.00	0.00	0.00	1000	60
HLWM1C	5.63	4.96	4.90	1000	60

Supplemental Table 1. Continued

Sample	Replicate 1	Replicate 2	Replicate 3	Filtered volume (mL)	Eluted volume (uL)
STA51A	0.00	0.00	0.00	500	25
STA51B	2.21	1.45	0.00	500	25
STA51C	0.00	0.00	0.00	500	25
STA52A	0.00	0.00	0.00	1000	25
STA52B	0.00	0.00	0.00	1000	25
STA52C	0.00	0.00	0.00	500	25
ELV1A	0.00	0.00	0.00	1000	60
ELV1B	0.00	0.00	0.00	1000	60
ELV1C	0.00	0.00	0.00	1000	60
ELV2A	15.72	13.80	6.99	1000	25
ELV2B	16.75	15.12	8.18	250	60
ELV2C	8.06	7.12	5.79	1000	60
ELV3A	54.74	42.18	37.93	1000	25
ELV3B	1.05	0.00	0.00	1000	60
ELV3C	177.71	160.61	145.40	1000	60
NOS1A	14.24	13.98	11.89	500	25
NOS1B	4.79	3.04	1.77	1000	60
NOS1C	16.06	13.31	9.15	500	60
NOS2A	2.06	1.45	0.00	1000	25
NOS2B	1.57	0.00	0.00	1000	60
NOS2C	1.36	0.00	0.00	700	60
NOS3A	10.03	3.02	3.00	1000	25
NOS3B	1.57	0.00	0.00	550	60
NOS3C	3.57	3.38	1.96	600	60
SWP1A	1.69	0.00	0.00	1000	25
SWP1B	5.72	2.69	1.53	500	60
SWP1C	0.00	0.00	0.00	750	25
SWP2A	0.00	0.00	0.00	1000	25
SWP2B	0.00	0.00	0.00	1000	25
SWP2C	0.00	0.00	0.00	500	25
SWP3A	59.88	58.52	46.30	500	25
SWP3B	1.06	0.00	0.00	1000	60
SWP3C	60.76	45.32	42.69	1000	60
