# Supplementary material for: Environmental DNA (eDNA) Sampling Improves Occurrence and Detection Estimates of Invasive Burmese Pythons
Source: PLoS One. 2015 Apr 15;10(4):e0121655. doi: 10.1371/journal.pone.0121655 (PMC4398459; doi:10.1371/journal.pone.0121655)
Supplement: S2 Table — Posterior mean and 95% credible interval (CI) are given for each parameter of the occupancy model. A uniform prior distribution was assumed. Average conditional probability of occurrence of Burmese python eDNA in a single sample (θ¯). Cumulative probability of detecting eDNA in three qPCR replicates (p*). BDB, Bird Drive Basin; DE, Deering Estates; ENP, Everglades National Park; HLWM, Holey Lands Wildlife Management Area; STA, Stormwater Treatment Area 5; and radiotagged snakes: ELV, Elvis; NOS, Noosa; and SWP, Sweet Pea. (PDF) [file pone.0121655.s005.pdf]

Supporting Information Table S2. Regional Bayesian estimates of occurrence (*ψ*) and detection (*p*) probabilities of *Python molurus bivittatus* environmental DNA. Posterior mean and 95% credible interval (CI) are given for each parameter of the occupancy model. A uniform prior distribution was assumed. Cumulative probability of detecting eDNA in three qPCR replicates is denoted by p^*. Average conditional probability of occurrence of python eDNA in a single eDNA sample is denoted ($\bar{\theta}$). BDB, Bird Drive Basin; DE, Deering Estates; ENP, Everglades National Park; HLWM, Holey Lands Wildlife Management Area; STA, Stormwater Treatment Area 5; and radiotagged snakes: ELV, Elvis; NOS, Noosa; and SWP, Sweet Pea.

Region	ψ	95% CI	$\bar{\theta}$	95% CI	p	95% CI	p^*	95% CI
BDB	0.66	0.22-0.98	0.55	0.25-0.87	0.46	0.18-0.77	0.80	0.45-0.99
DE	0.80	0.40-0.99	0.69	0.46-0.90	0.58	0.37-0.78	0.91	0.75-0.99
ENP	0.80	0.40-0.99	0.54	0.32-0.76	0.75	0.51-0.93	0.98	0.89-1.00
STA	0.56	0.11-0.97	0.45	0.08-0.93	0.51	0.12-0.92	0.81	0.31-1.00
ELV	0.68	0.23-0.99	0.71	0.44-0.96	0.85	0.67-0.97	0.99	0.96-1.00
NOS	0.80	0.40-0.99	0.80	0.59-0.95	0.72	0.55-0.87	0.97	0.91-1.00
SWP	0.63	0.21-0.97	0.68	0.40-0.93	0.69	0.45-0.89	0.96	0.83-1.00
